# Auxiliary diagnostic value of tumor biomarkers in pleural fluid for lung cancer-associated malignant pleural effusion

**DOI:** 10.1186/s12931-020-01557-z

**Published:** 2020-10-29

**Authors:** Hai Zhang, Changhui Li, Fang Hu, Xueyan Zhang, Yinchen Shen, Yuqing Chen, Feng Li

**Affiliations:** Department of Pulmonary and Critical Care Medicine, Shanghai Chest Hospital, Shanghai Jiao Tong University, NO. 241, West Huaihai Road, Shanghai, 200030 China

**Keywords:** Tumor markers, Lung cancer, Pleural effusion, Serum, CEA

## Abstract

**Background:**

Pleural effusion (PE) can be divided into benign pleural effusion (BPE) and malignant pleural effusion (MPE). There is no consensus on the identification of lung cancer-associated MPE using the optimal cut-off levels from five common tumor biomarkers (CEA, CYFRA 21-1, CA125, SCC-Ag, and NSE). Therefore, we aimed to find indicators for the auxiliary diagnosis of lung cancer-associated MPE by analyzing and then validating the optimal threshold levels of these biomarkers in pleural fluid (PF) and serum, as well as the PF/serum ratio.

**Patients and method:**

The study has two sets of patients, i.e. the training set and the test set. In the training set, 348 patients with PE, between January 1, 2016 and December 31, 2017, were divided into BPE and MPE based on the cytological diagnosis. Subsequently, the optimal cut-off levels of tumor biomarkers were analyzed. In the test set, the diagnostic compliance rate was verified with 271 patients with PE from January 1, 2018 to July 31, 2019 to evaluate the auxiliary diagnostic value of the aforementioned indicators.

**Result:**

In the training set, PF CEA at the cut-off value of 5.23 ng/ml was the most effective indicator for MPE compared with other tumor biomarkers (all *p* < 0.001). In the test set, PF CEA at the cut-off value of 5.23 ng/ml showed the highest sensitivity, specificity and accuracy, positive and negative predictive value among other tumor biomarkers, which were 99.0%, 69.1%, 91.6%, 90.7%, and 95.9%, respectively.

**Conclusion:**

PF CEA at the cut-off level of 5.23 ng/ml was the most effective indicator for identifying lung cancer-associated MPE among the five common tumor biomarkers.

## Background

Pleural effusion (PE) can be roughly divided into benign pleural effusion (BPE) and malignant pleural effusion (MPE). MPE is a common complication of lung cancer, and patients are usually diagnosed with stage IV [1, 2]. Approximately 15% of lung cancer patients have PE at the time of initial diagnosis, and 50% of patients have PE during the course of lung cancer [3]. The presence of MPE usually indicates a poor prognosis. The median survival of patients with untreated MPE is only 4 months [1]. MPE can also significantly reduce patients' quality of life [4]. Therefore, the occurrence of MPE in lung cancer should be identified as soon as possible.

The most common way to distinguish between MPE and BPE depends on the cytological analysis of pleural fluid (PF). This method is 100% specific, but the sensitivity is only 60% [5]. When PE cytology is not indicative, the diagnosis can be confirmed by pleural biopsy or thoracoscopy [6, 7]. However, a previous study has shown that pleural biopsy can only diagnose 7% of patients with negative PF cytology [8]. In addition, the area for pleural biopsy to enter the thoracic cavity is limited, and the malignant pleura may be scattered in hard-to-reach areas or confined to the surface of the visceral pleura. Hence, not all patients are suitable for this invasive surgery [9].Therefore, when the nature of PE cannot be determined by cytology, tumor biomarkers in PF are considered as a viable option to distinguish between MPE and BPE.

Conventional tumor biomarkers include carcinoembryonic antigen (CEA), cytokeratin 19 fragment (CYFRA 21-1), squamous cells cancer (SCC) antigen, neuron-specific enolase (NSE), and cancer antigen 125 (CA125) [10]. To our knowledge, no consensus on the optimal cut-off level for these biomarkers in the auxiliary identification of lung cancer-associated MPE. Therefore, in this study, we analyzed and then verified the optimal cut-off level of such biomarkers in PF and serum, as well as the PF/serum ratio for the auxiliary diagnosis of lung cancer-associated MPE.

## Patients and methods

The clinical data of 619 patients with PE admitted to the Department of Pulmonary and Critical Care Medicine of Shanghai Chest Hospital, Shanghai Jiao Tong University from January 01, 2016 to July 31, 2019 were selected and analyzed retrospectively. Patients were divided into the training set and the test set, i.e. patients from January 01, 2016 to December 31, 2017 formed the training set, and the patients from January 1, 2018 to July 31, 2019 formed the test set.

The MPE or BPE was diagnosed based on the cytology and follow-up (at least 6 months). Both the training set and test set followed the same diagnostic criteria for lung cancer-associated MPE, which included (1) lung cancer diagnosed by the cytological diagnosis of PF or other ways such as bronchoscopy; (2) no other malignancies. The criteria for BPE were as follow: (1) no tumor cells were found in PF; (2) the PE vanished after etiological treatment and thoracentesis, and then did not recur; and (3) no cancer diagnosis was established during the follow-up.

The following patients were excluded: (1) PE caused by mesothelioma (some studies demonstrated that CEA did not increase significantly when mesothelioma caused PF [10, 11]); (2) MPE caused by other organ’s tumor; (3) no lung cancer cells found in PF of patients with confirmed lung cancer; and (4) the pathological type undetermined by all means.

### Study design

Patient medical records and follow-up data were collected and analyzed. The detailed data were as follows: age, sex, smoking history, Eastern Cooperative Oncology Group (ECOG) performance status (PS), level of tumor biomarkers (CEA, CYFRA 21-1, CA125, SCC-Ag, and NSE) in PF and serum, and the PF/serum ratios. Patient information was classified based on age (< 60 years or ≥ 60 years), sex (male or female), smoking history (yes or no), and ECOG PS (0–1 or 2–3). The patients were divided into the MPE group and the BPE group by cytological diagnosis. The pathological types of patients with MPE were also recorded. This study was approved by the Ethics Committee and the Institutional Review Board and was conducted in accordance with the Helsinki Declaration. All patients consented to the use of their data for research and signed written informed consent before the collection of information.

### Detection of tumor biomarkers in PF and serum

All enrolled subjects underwent standard thoracentesis within 24 h of admission. PF and serum samples were collected from patients and then transported to the Department of Laboratory Medicine within 30 min after collection. All measurements were performed following the manufacturer’s instruction. The Tellgen Super Multiplex Immunoassay (TESMI™) Tumor Marker Panel (5-markers) (Tellgen Corporation, Shanghai, China), based on the TESMI system technology, was used to detect the concentration of tumor biomarkers in the samples. The upper limit of normal CEA, CYFRA 21-1, CA125, SCC-Ag and NSE in serum was defined as a cut-off value of 5.0 ng/ml, 5.0 ng/ml, 35.0 U/ml, 1.5 ng/ml, and 25.0 ng/ml, respectively.

### Statistical analysis

The data from the two groups were analyzed using the *χ*^2^ test. In the training set, the receiver operating characteristic (ROC) curve was used to determine the best cut-off levels of each tumor biomarker, and the area under the curve (AUC) of each tumor biomarker was calculated. The threshold was selected based on the highest diagnostic efficacy having achieved equilibrium between sensitivity and specificity by using Youden’s index. The P value of each biomarker was obtained by comparing the difference of each biomarker’s level between BPE and MPE. Furthermore, the effective tumor biomarkers were defined as biomarkers with an AUC value greater than 0.7 [13]. In test set, the sensitivity, specificity and accuracy, positive and negative predictive values of the effective tumor biomarkers for lung cancer-associated MPE were calculated. SPSS 23.0 software was used for statistical analysis and *P* < 0.05 indicated statistical significance.

## Results

### Patient characteristics

**Fig. 1:**
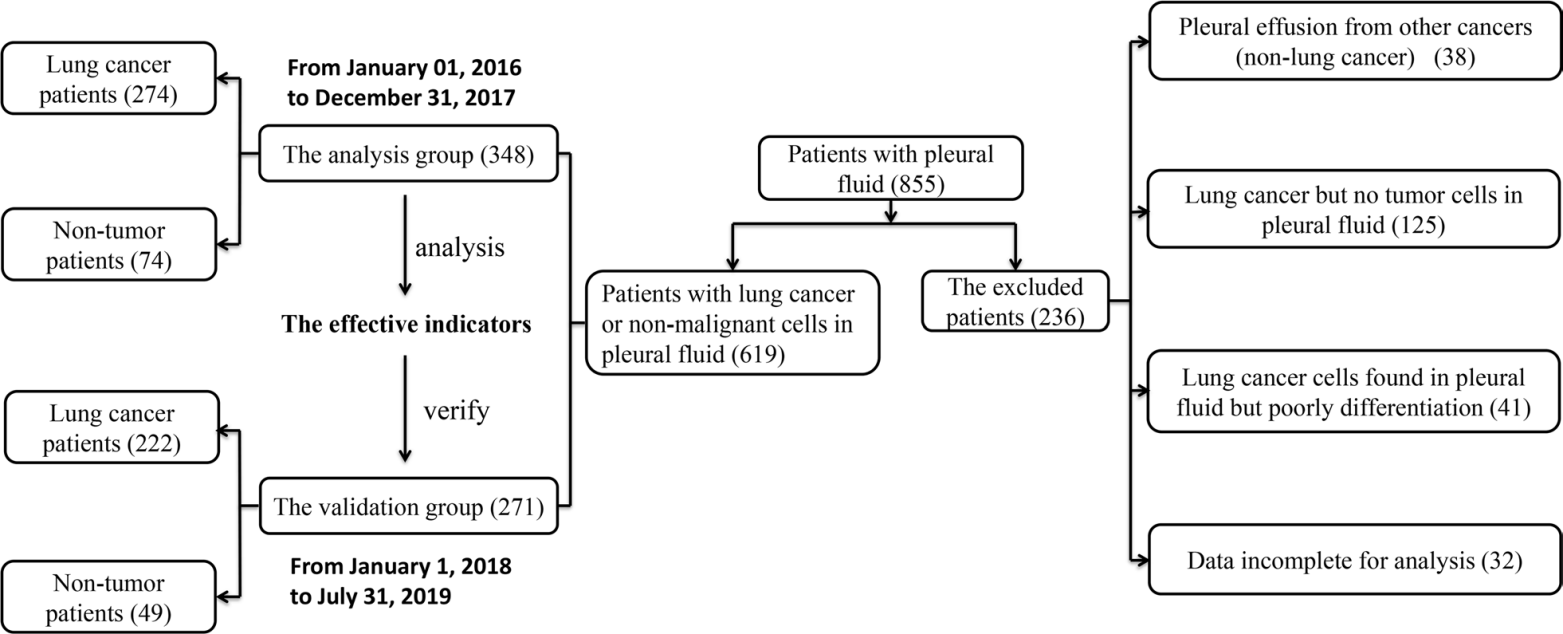
The patients’ selection flowchart

A total of 855 patients were diagnosed with PE. Among them, 236 patients who did not meet the enrollment criteria were therefore excluded from the study. Finally, 619 eligible patients were included and reviewed in this study. The patients’ selection flowchart is shown in Fig. 1. 348 patients from January 1, 2016 to December 31, 2017 formed the training set (274 patients with lung cancer and 74 patients with benign lung diseases), and 271 patients from January 1, 2018 to July 31, 2019 formed the test set (222 patients with lung cancer and 49 patients with benign lung diseases). Whether in the training set or the test set, no difference was observed in age, sex, smoking history, and ECOG PS between the MPE group and the BPE group. The baseline of patient characteristics is listed in Table 1.

**Table 1:** Comparison of baseline characteristics between the MPE group and the BPE group in both the training set and test set

Characteristics	The training set			The test set			
	MPE	BPE	*P*	MPE		BPE	*P*
Age							
< 60	112	39	0.069	84		24	0.149
≥ 60	162	35		138		25	
Gender							
Male	144	46	0.141	130		34	0.160
Female	130	28		92		15	
Smoking history							
Yes	100	31	0.395	97		25	0.351
ECOG-PS							
0–1	261	67	0.122	214		44	0.500
2–3	13	7		8		5	
Cancer type^a^							
Adenocarcinoma	261	–		210		–	
Squamous cell carcinoma	5	–		2		–	
Small cell lung cancer	8	–		10		–	
No-lung cancer type^b^							
Tuberculosis	–	28		–		24	
Bacterial infections	–	13		–		8	
Other diseases	–	33			–	17	

MPE, malignant pleural effusion; BPE, benign pleural effusion; EGFR, epidermal growth factor receptor; ECOG, eastern cooperative oncology group; PS, performance status

^a^The value of % was calculated among the number of cancer type

^b^The value of % was calculated among the number of no-lung cancer type

The diagnostic performance of tumor biomarkers for lung cancer-associated MPE in the training set.

ROC analysis was performed to determine the cut-off levels of sensitivity and specificity of each tumor biomarker. The effective tumor biomarkers were defined as biomarkers with an AUC value greater than 0.7. The detailed ROC analysis of each tumor biomarker is listed in Table 2. The diagnostic values of effective indicators were as follows: when the cut-off value of PF CEA was 5.23 ng/ml, the sensitivity and specificity were 89.8% and 98.6%, respectively, and with an AUC of 0.978. At the cut-off value of 2.7 ng/ml in serum CEA, the sensitivity and specificity were 81.4% and 86.5%, respectively, and with an AUC of 0.900. Moreover, the sensitivity and specificity of the PF/serum ratio of CEA were 82.5% and 86.5%, respectively, with an AUC of 0.896 at the cut-off value of 1.365. The sensitivity and specificity of PF CYFRA 21-1 and serum CYFRA21-1 were 67.9% and 90.5%, and 77.7% and 73.0%, with an AUC of 0.853 and 0.812, respectively, when the cut-off value was set at 31.39 ng/ml and 2.09 ng/ml, respectively. The other tumor biomarkers, including SCC-Ag, NSE and CA125, did not have an AUC of more than 0.7.

**Table 2:** Diagnostic performance of tumor biomarkers in pleural fluid of lung cancer-associated MPE

	Sensitivity (%)	Specificity (%)	Criterion	AUC	*P*
*CEA*					
PF	89.8	98.6	5.23 ng/ml	0.978	< 0.001
Serum	81.4	86.5	2.7 ng/ml	0.900	< 0.001
PF/Serum	82.5	86.5	1.365	0.896	< 0.001
*CYFRA 21-1*					
PF	67.9	90.5	31.39 ng/ml	0.853	< 0.001
Serum	77.7	73.0	2.09 ng/ml	0.812	< 0.001
PF/Serum	80.1	45.9	4.38	0.675	< 0.001
*SCC-Ag*					
PF	69.3	54.1	1.9 ng/ml	0.609	0.004
Serum	61.7	56.8	0.6 ng/ml	0.610	0.002
PF/Serum	70.8	60.8	2.41	0.659	< 0.001
*NSE*					
PF	69.0	45.9	8.65 ng/ml	0.600	0.007
Serum	50.3	78.4	18.78 ng/ml	0.667	< 0.001
PF/Serum	16.8	93.2	2.41	0.530	0.426
*CA125*					
PF	61.2	75.7	698.2 ng/ml	0.698	< 0.001
Serum	32.8	82.4	126 ng/ml	0.558	0.098
PF/Serum	30.8	90.5	11.22	0.599	0.005

*CEA* carcinoembryonic antigen, *CYFRA 21-1* cytokeratin 19 fragment, *SCC-Ag* squamous cells cancer antigen, *NSE* neuron-specific enolase, *CA125* cancer antigen 125, *PF* pleural fluid, *PF/Serum* PF value divided by serum value

### Comparison of tumor biomarker with ROC curves

**Fig. 2:**
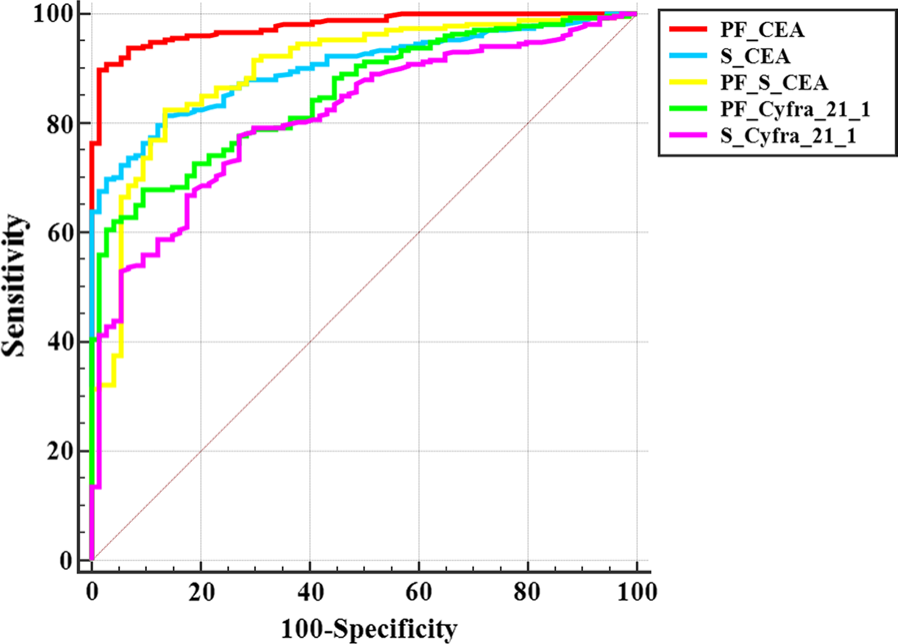
The ROC curves of the tumor biomarkers to distinguish between MPE and BPE

Comparison results of tumor biomarkers are listed in Table 3. PF CEA showed the most discriminative ability (all *P* < 0.001). Furthermore, the ROC curves of PF CEA, serum CEA, PF/serum CEA, PF CYFRA 21-1, and serum CYFRA 21-1 to identify the most effective biomarkers are shown in Fig. 2. As a result, PF CEA was the most discriminative biomarker for lung cancer-associated MPE.

**Table 3:** Comparison of tumor biomarkers with an AUC greater than 0.7

	PF CEA	Serum CEA	PF/serum CEA	PF CYFRA 21-1
Serum CEA	< 0.001	–	–	–
PF/serum CEA	< 0.001	0.885	–	–
PF CYFRA 21-1	< 0.001	0.067	0.132	–
Serum CYFRA 21-1	< 0.001	0.016	0.018	0.188

*CEA* carcinoembryonic antigen, *CYFRA 21-1* cytokeratin 19 fragment, *PF* pleural fluid, *PF/serum* PF value divided by serum value

The diagnostic performance of tumor biomarkers for lung cancer-associated MPE in the test set.

**Fig. 3:**
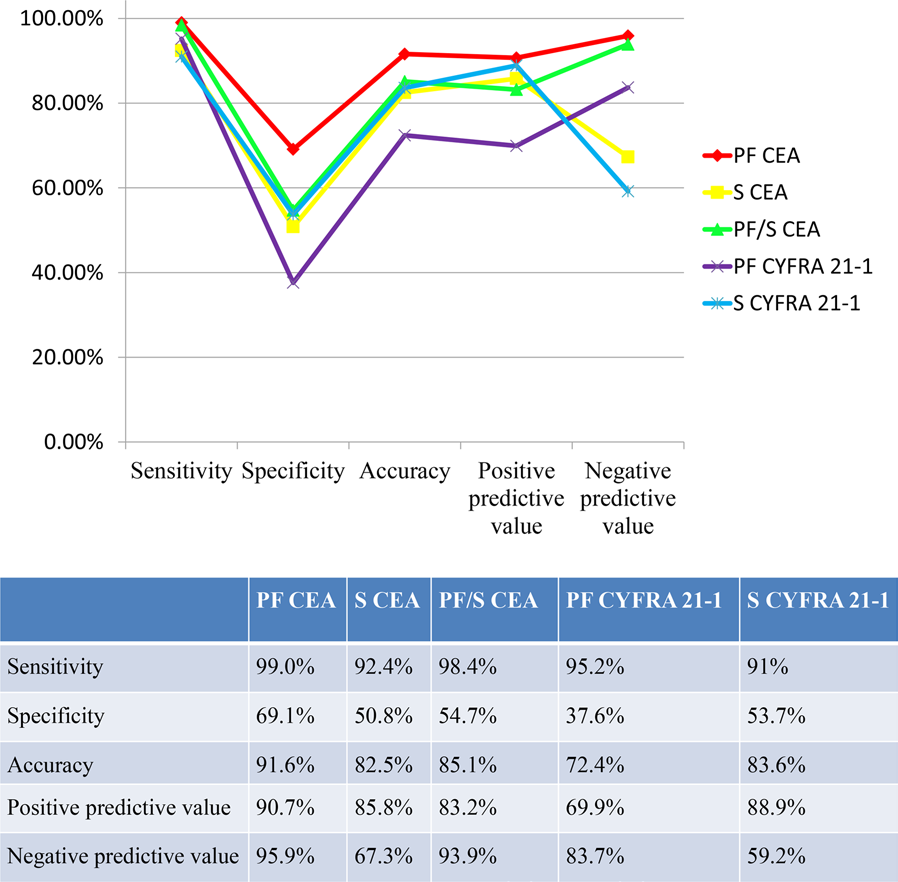
The verification results among the effective tumor biomarkers

The test set consisted of 271 patients (222 patients with lung cancer and 49 patients with non-lung cancer). The classification results are listed in Additional file 1. Table S1. And the verification results were shown in Fig. 3. Compared with other effective indicators, PF CEA at 5.23 ng/ml showed the highest diagnosis rate, and the sensitivity, specificity, accuracy, positive and negative predictive values were 99.0%, 69.1%, 91.6%, 90.7%, and 95.9%, respectively.

## Discussion

This study examined the best cut-off levels of five common lung cancer biomarkers (CEA, CYFRA 21-1, CA125, SCC-Ag, and NSE) from PF, serum and the PF/serum ratio value for identifying lung cancer-associated MPE. It demonstrated that PF CEA was the most effective biomarker to identify lung cancer-associated MPE at the cut-off value of 5.23 ng/ml (sensitivity at 89.8%, specificity at 98.6%) in the training set. Furthermore, in the test set, PF CEA at 5.23 ng/ml showed the highest sensitivity, specificity, and accuracy, positive and negative predictive values for lung cancer-associated MPE when compared with other tumor biomarkers. Therefore, it was concluded that PF CEA at the cut-off level of 5.23 ng/ml may be the most effective indicator for identifying lung cancer-associated MPE.

PE is a common and important complication, caused by many diseases, especially malignant tumors [14]. It is important to understand the etiology of PE, especially to distinguish between BPE and MPE [15]. Thoracentesis, as well as cytology and histology, is a preliminary diagnostic method of PE [6, 7]. However, these common methods sometimes can produce false negatives. Therefore, auxiliary indicators are needed to improve the accuracy of diagnosis. In recent years, tumor biomarkers have been found to be important in the differential diagnosis of PE [16]. Among these parameters, CEA, CYFRA 21-1, CA125, SCC-Ag, and NSE were found to have a more significant diagnostic value than others [10]. Some studies have shown that the tumor biomarkers in serum had good diagnostic significance, while other studies have shown that tumor biomarkers in PF had better identification significance than that in serum [17–19]. Even some studies have shown that PF/serum ratio can be used as a good diagnostic indicator [20]. However, it was unclear which biomarkers were the most effective indicator in identifying MPE. In this study, the PF value, serum value, and PF/serum ratio of different tumor biomarkers were compared to find the best diagnostic indicator. The study demonstrated that CEA and CYFRA 21-1, especially PF CEA, serum CEA, PF/serum CEA, PF CYFRA 21-1 and serum CYFRA 21-1, had better auxiliary diagnostic significance than other biomarkers in defining lung cancer-associated MPE.

CEA, a glycoprotein involved in cell adhesion, is the earliest fetal embryo antigen, and is composed of a family of cell surface glycoproteins. It is usually produced during fetal development, but its production stops before birth. Therefore, it is usually not found in the blood of healthy adults [17, 21]. Currently, CEA in serum is the most convenient method to be used as a potential prognostic marker for lung cancer [21]. CYFRA 21-1 is a polypeptide that recognizes a soluble cytokeratin 19 fragment, and cytokeratin 19 is an acidic type I cytokeratin found in lung cancer cells [3, 22]. Therefore, CEA and CYFRA 21-1 are vital in the diagnosis of lung cancer. Some studies demonstrated that serum CEA and serum CYFRA 21-1 levels increased in patients with lung cancer [23, 24]. Also, CEA and CYFRA 21-1 levels increased in MPE patients when compared with BPE patients [19]. Although the aforementioned indicators have good accuracies, the tumor biomarkers with the highest accuracy are not known and needed to be determined.

Therefore, this study further compared the value of different tumor biomarkers in the differential diagnosis of MPE. It showed that CEA was more indicative of MPE than other tumor biomarkers. More importantly, PF CEA had a better indicative value than serum CEA. One possible mechanism is that the tumor cells metastasize to the pleura through the direct invasion of the pleura or the blood [25, 26]. The tumor that invaded the pleura secreted tumor biomarkers into the pleural cavity, or the tumor biomarkers were released into the blood and were then diluted [27]. In addition, tumor cells might block lymphatic drainage and reduce tumor indicators into the blood, and therefore tumor biomarkers were concentrated in the chest cavity [28]. Consequently, the levels and positive rates of tumor biomarkers in PF were significantly higher than those in serum which was observed in the study.

Examinations were conducted in the test set to verify the validity of the results from the training set. Tumor biomarkers with an AUC greater than 0.7 were compared. This study confirmed the training set results. Compared with other tumor biomarkers, PF CEA with a cut-off point at 5.23 ng/ml demonstrated the highest diagnosis rate, and the highest sensitivity, specificity, and accuracy, positive and negative predictive values for lung cancer-associated MPE. Therefore, the CEA cut-off level in PF at 5.23 ng/ml is the best indicator for identifying lung cancer-associated MPE. The normal upper limit of CEA in serum is 5 ng/ml, however, a slight increase of CEA in PF, that is 5.23 ng/ml, was indicative of MPE. This was a very significant result of the present study.

The study has several limitations. First, this is a single retrospective study and a non-randomized study. Second, the study patients were mainly patients with MPE related to lung cancer. For the accuracy of the indicators, patients diagnosed with lung cancer but no tumor cells in the PF were excluded. Last, as the majority of lung cancer types are adenocarcinoma in the study, the results may be suitable for lung adenocarcinoma-associated MPE. More samples from other types of lung cancer are needed for further study in the future.

## Conclusion

In conclusion, a comparison of tumor indicators in PF and serum, and the PF/serum ratio of CYFRA 21-1, CA125, SCC-Ag and NSE revealed that PF CEA at a cut-off level of 5.23 ng/ml was the most effective indicator for identifying lung cancer-associated MPE.

## Supplementary information

**Additional file 1: Table S1.** Classification results in the test set.

## Data Availability

The participating patients only agreed to use the information for this study and refused to share it.

## Abbreviations

PE: Pleural effusion
MPE: Malignant pleural effusion
BPE: Benign pleural effusion
PF: Pleural fluid
CEA: Carcinoembryonic antigen
CYFRA 21-1: Cytokeratin 19 fragment
SCC: Squamous cells cancer antigen
NSE: Neuron-specific enolase
CA125: Cancer antigen 125
ECOG: Eastern Cooperative Oncology Group
PS: Performance status
PF/S: PF value divided by serum value
ROC: Receiver operating characteristic
AUC: Area under the curve

## References

[1] Wang S, Tian S, Li Y, Zhan N, Guo Y, Liu Y (2020). Development and validation of a novel scoring system developed from a nomogram to identify malignant pleural effusion. EBioMedicine..

[2] Thompson JC, Fan R, Black T, Yu GH, Savitch SL, Chien A (2019). Measurement and immunophenotyping of pleural fluid EpCAM-positive cells and clusters for the management of non-small cell lung cancer patients. Lung Cancer.

[3] Feng M, Zhu J, Liang L, Zeng N, Wu Y, Wan C (2017). Diagnostic value of tumor markers for lung adenocarcinoma-associated malignant pleural effusion: a validation study and meta-analysis. Int J Clin Oncol.

[4] Thomas JM, Musani AI (2013). Malignant pleural effusions: a review. Clin Chest Med.

[5] Hooper C, Lee YC, Maskell N, BTS Pleural Guideline Group (2010). Investigation of a unilateral pleural effusion in adults: British Thoracic Society Pleural Disease Guideline 2010. Thorax.

[6] Maskell NA, Gleeson FV, Davies RJ (2003). Standard pleural biopsy versus CT-guided cutting-needle biopsy for diagnosis of malignant disease in pleural effusions: a randomised controlled trial. Lancet.

[7] Menzies R, Charbonneau M (1991). Thoracoscopy for the diagnosis of pleural disease. Ann Intern Med.

[8] Lombardi G, Zustovich F, Nicoletto MO, Donach M, Artioli G, Pastorelli D (2010). Diagnosis and treatment of malignant pleural effusion: a systematic literature review and new approaches. Am J Clin Oncol.

[9] Noppen M (2010). The utility of thoracoscopy in the diagnosis and management of pleural disease. Semin Respir Crit Care Med.

[10] Ferrer J, Villarino MA, Encabo G, Felip E, Bermejo B, Vilà S (1999). Diagnostic utility of CYFRA 21-1, carcinoembryonic antigen, CA 125, neuron specific enolase, and squamous cell antigen level determinations in the serum and pleural fluid of patients with pleural effusions. Cancer.

[11] Mezger J, Calavrezos A, Drings P, Gatzemeier U, Kaukel E, Konietzko N (1994). Value of serum and effusion fluid CEA levels for distinguishing between diffuse malignant mesothelioma and carcinomatous pleural metastases. Lung.

[12] Whitaker D, Shilkin KB, Stuckey M, Nieuwhof WN (1986). Pleural fluid CEA levels in the diagnosis of malignant mesothelioma. Pathology.

[13] Gao Yu, Kalbasi A, Hsu W, Ruan D, Jie Fu, Shao J (2020). Treatment effect prediction for sarcoma patients treated with preoperative radiotherapy using radiomics features from longitudinal diffusion-weighted MRI. Phys Med Biol..

[14] Perricone G, Airoldi A, Vangeli M (2018). Pleural disease. N Engl J Med.

[15] Porcel JM, Esquerda A, Vives M, Bielsa S (2014). Etiology of pleural effusions: analysis of more than 3,000 consecutive thoracenteses. Arch Bronconeumol.

[16] Porcel JM (2018). Biomarkers in the diagnosis of pleural diseases: a 2018 update. Ther Adv Respir Dis.

[17] Tozzoli R, Basso SM, D'Aurizio F, Metus P, Lumachi F (2016). Evaluation of predictive value of pleural CEA in patients with pleural effusions and histological findings: a prospective study and literature review. Clin Biochem.

[18] Lee JH, Chang JH (2005). Diagnostic utility of serum and pleural fluid carcinoembryonic antigen, neuron-specific enolase, and cytokeratin 19 fragments in patients with effusions from primary lung cancer. Chest.

[19] Hackbarth JS, Murata K, Reilly WM, Algeciras-Schimnich A (2010). Performance of CEA and CA19-9 in identifying pleural effusions caused by specific malignancies. Clin Biochem.

[20] Hackner K, Errhalt P, Handzhiev S (2019). Ratio of carcinoembryonic antigen in pleural fluid and serum for the diagnosis of malignant pleural effusion. Ther Adv Med Oncol.

[21] Grunnet M, Sorensen JB (2012). Carcinoembryonic antigen (CEA) as tumor marker in lung cancer. Lung Cancer.

[22] Fu L, Wang R, Yin L, Shang X, Zhang R, Zhang P (2019). CYFRA21-1 tests in the diagnosis of non-small cell lung cancer: a meta-analysis. Int J Biol Markers.

[23] Wang L, Wang D, Zheng G (2016). Clinical evaluation and therapeutic monitoring value of serum tumor markers in lung cancer. Int J Biol Markers.

[24] Okamura K, Takayama K, Izumi M, Harada T, Furuyama K, Nakanishi Y (2013). Diagnostic value of CEA and CYFRA 21–1 tumor markers in primary lung cancer. Lung Cancer.

[25] Rodrîguez-Panadero F, Borderas Naranjo F, López MJ (1989). Pleural metastatic tumours and effusions. Frequency and pathogenic mechanisms in a post-mortem series. Eur Respir J..

[26] Meyer PC (1966). Metastatic carcinoma of the pleura. Thorax.

[27] Chen Z, Wang Y, Fang M (2020). Analysis of tumor markers in pleural effusion and serum to verify the correlations between serum tumor markers and tumor size, TNM stage of lung adenocarcinoma. Cancer Med.

[28] Psallidas I, Kalomenidis I, Porcel JM, Robinson BW, Stathopoulos GT (2016). Malignant pleural effusion: from bench to bedside. Eur Respir Rev.

