# Supplementary material for: Auxiliary diagnostic value of tumor biomarkers in pleural fluid for lung cancer-associated malignant pleural effusion
Source: Respir Res. 2020 Oct 29;21:284. doi: 10.1186/s12931-020-01557-z (PMC7596935; doi:10.1186/s12931-020-01557-z)
Supplement: Supplementary file 1 — Additional file 1: Table S1. Classification results in the test set. [file 12931_2020_1557_MOESM1_ESM.docx]

Additional Table 1. Classification results in the test set

|  | ≤ the analysis value | > the analysis value |
| --- | --- | --- |
|  | No. (%) | No. (%) |
| PF CEA |  |  |
| Cut-off | ≤5.23 | >5.23 |
| MPE | 21 (7.6%) | 205 (74.6%) |
| BPE | 47 (17.1%) | 2 (0.7%) |
| Serum CEA |  |  |
| Cut-off | ≤2.7 | >2.7 |
| MPE | 32 (11.6%) | 194 (70.5%) |
| BPE | 33 (12.0%) | 16 (5.8%) |
| PF/Serum CEA |  |  |
| Cut-off | ≤1.365 | >1.365 |
| MPE | 38 (13.8%) | 188 (68.4%) |
| BPE | 46 (16.7%) | 3 (1.1%) |
| PF CYFRA 21-1 |  |  |
| Cut-off | ≤31.39 | >31.39 |
| MPE | 68 (24.7%) | 158 (57.5%) |
| BPE | 41 (14.9%) | 8 (2.9%) |
| Serum CYFRA 21-1 |  |  |
| Cut-off | ≤2.09 | >2.09 |
| MPE | 25 (9.1%) | 201 (73.1%) |
| BPE | 29 (10.5%) | 20 (7.3%) |

Abbreviation: MPE, malignant pleural effusion; BPE, benign pleural effusion; PF, pleural fluid; CEA, carcinoembryonic antigen; CYFRA 21-1, cytokeratin 19 fragment; PF/Serum, PF value divided by serum value.
